# Structural alteration of lung parenchyma in patients with NF1: a phenotyping study using multidetector computed tomography (MDCT)

**DOI:** 10.1186/s13023-021-01672-0

**Published:** 2021-01-14

**Authors:** Maxim Avanesov, Lennart Well, Azien Laqmani, Thorsten Derlin, Vincent M. Riccardi, Gerhard Adam, Victor-Felix Mautner, Johannes Salamon

**Affiliations:** 1 Department of Diagnostic and Interventional Radiology and Nuclear Medicine, University Medical Center Hamburg-Eppendorf, Hamburg, Germany; 2 Department of Nuclear Medicine, Hannover Medical School, Hannover, Germany; 3 The Neurofibromatosis Institute, La Crescenta, CA USA; 4 Clinic and Polyclinic for Neurology, Neurofibromatosis Outpatient Clinic, UKE, Hamburg, Germany

**Keywords:** Neurofibromatosis 1, Pulmonary cysts, Pulmonary nodules, Emphysema, MDCT, Malignant peripheral nerve sheath tumor

## Abstract

**Background:**

Diffuse interstitial lung disease have been described in Neurofibromatosis type 1 (NF1), but its diversity and prevalence remain unknown. The aim of this study was to assess the prevalence and characteristics of (NF1)-associated lung manifestations in a large single-center study using multidetector computed tomography (MDCT) and to evaluate the smoking history, patients’ age, genetics, and the presence of malignant peripheral nerve sheath tumors (MPNST) as potential influencing factors for lung pathologies.

**Methods:**

In this retrospective study, 71 patients with NF1 were evaluated for the presence of distinctive lung manifestations like reticulations, consolidations, type of emphysema, pulmonary nodules and cysts. All patients underwent F-18-FDG PET/CT scans, which were reviewed by two experienced radiologists in consensus. Patients’ subgroups were formed based on their smoking history (current smokers/previous smokers/never smokers), age (< 12 years, 12–18 years, > 18 years), and presence of MPNST (MPNST/no MPNST). In 57 patients (80%), genetic analysis of sequences coding for the neurofibromin on chromosome 17 was performed, which was correlated with different lung pathologies.

**Results:**

Among all NF1 patients (33 ± 14 years, 56% females), 17 patients (24%) were current smokers and 62 patients (87%) were > 18 years old. Pulmonary cysts, nodules, and paraseptal emphysema were the most common pulmonary findings (35%, 32%, 30%). The presence of pulmonary metastases, MPNST and centrilobular emphysema was associated with smoking. Cysts were observed only in adults, whereas no significant correlation between age and all other pulmonary findings was found (*p* > 0.05). Presence of MPNST was accompanied by higher rates of intrapulmonary nodules and pulmonary metastasis. Neither the presence nor absence of any of the specific gene mutations was associated with any particular lung pathology (*p* > 0.05).

**Conclusions:**

All pulmonary findings in NF1 patients occurred independently from specific mutation subtypes, suggesting that many NF1 mutations can cause various pulmonary pathologies. The presence of pulmonary metastases, MPNST and centrilobular emphysema was associated with smoking, indicating the value of smoking secession or the advice not to start smoking in NF1 patients as preventive strategy for clinicians. For screening of pulmonary manifestations in NF1 patients, an MDCT besides medical history and physical examination is mandatory in clinical routine.

## Introduction

Neurofibromatosis type 1 (NF1) is a rare hereditary tumor predisposition syndrome caused by an autosomal-dominant mutation in the NF1 tumor suppressor gene [1]. NF1 has a prevalence of 1 in 3000. 30–50% of cases are caused by de-novo-mutation with no prior family history of the disease [2].

Individuals with NF1 may develop a variety of musculoskeletal thoracic manifestations including thoracic kyphoscoliosis, ribbon deformity of the ribs, meningoceles as well as a set of benign and malignant nerve sheath tumors such as cutaneous and subcutaneous neurofibromas, plexiform Neurofibromas and malignant peripheral nerve sheath tumors of the chest wall and mediastinum. Interstitial lung disease (ILD) with bullae formation is frequently reported [3] and is assumed to be caused by genetic predisposition [4–6], especially pulmonary fibrosis [4, 6].

However, today’s knowledge of the prevalence and the characteristics of NF1-associated lung disease is mostly based on a low number of published case reports. The majority of previous studies used plain X-ray only [7]. The few studies using computed tomography (CT) had considerable small patient populations of eight [7] and six patients [8] and in two publications inhomogeneous CT study protocols were applied with a variety of different vendors [7, 9] and numbers of detector rows ranging from 1 to 64 rows [9].

Moreover, the association of the smoking history with pulmonary pathologies remains unclear [3, 8, 9]. While Oikonomou et al. [8] observed different manifestations of interstitial lung disease in non-smoking NF1 patients, Ueda et al. [9] found a strong association of the occurrence of emphysema with the smoking history of NF1 patients. Other possible confounders as age and the presence of malignant peripheral nerve sheath tumor (MPNST, indicating a high penetrance of the disease) on the occurrence of interstitial lung disease in NF1 patients have not yet been investigated.

Despite the fact, that more than 500 different mutations of the NF1 gene have been identified so far, only the in-frame deletion of Exon 17 and a whole NF1 gene deletion were associated with either solely pigmentary features of NF1 without plexiform neurofibromas or a more frequent and severe course of the disease including more cutaneous neurofibromas, frequent cognitive abnormalities, and a marfanoid habitus, respectively [10]. However, the association of specific mutation types with different pulmonary pathologies remains unknown.

Therefore, the aim of the present study was to assess different lung manifestations in NF1 patients in a large single-center cohort using a standardized MDCT protocol and to evaluate smoking history, patients’ age, specific genetic analysis of all NF1 exons and the presence of MPNST as potential influencing factors for lung pathologies.

## Materials and methods

### Patients

The study group included 71 patients with NF1 with benign (n = 43) or malignant PNSTs (n = 28) who underwent F-18-FDG PET/CT scans for exclusion or progress evaluation of MPNSTs between May 2006 and May 2018. The inclusion and exclusion criteria were as follows:

#### Inclusion criteria

1. Fulfillment of the National Institutes of Health (NIH) diagnostic criteria with genetic confirmation for NF1 [11].

2. Available F-18-FDG PET/CT scans with MDCT of the lungs and multiplanar reconstructions (MPR) of the lung window in the transversal, coronal and sagittal orientations.

#### Exclusion criteria

1. Inability or unwillingness to provide informed consent for the retrospective analysis of the data.

2. NF patients with unavailable/unknown history of smoking.

All NF1 patients were divided into subgroups based on their smoking history (current smoker, previous smoker, never smoker), their age (12 years, 12–18 years, > 18 years), and the presence of MPNST (MPNST vs. no MPNST). Current smokers included patients, who smoke regularly, while previous smokers consisted of patients, who have quitted smoking > 5 years ago. Never smokers didn’t smoke during their entire life. Patients at different ages were compared with each other as age is a known influencing factor for occurrence and progression of plexiform neurofibromas, gliomas, and musculoskeletal deformations [12, 13]. Therefore, an influence of age on the occurrence of pulmonary findings may also be assumed.

The study protocol was approved by our local IRB (Ethic committee of the medical chamber of Hamburg) and complied with the Declaration of Helsinki. All study patients had given written informed consent for the retrospective evaluation of their data. For children written informed consent was obtained from their legal guardians.

### PET/CT acquisition and image reconstruction

All patients underwent a F-18-FDG PET/CT (Gemini GLX10 PET/CT system with a 16 MDCT, Philips Medical Systems®, Best, The Netherlands). Imaging started with a low-dose CT of the whole body (120 kV, 80 mA, transaxial FOV 600 mm, no gap, collimation 10 × 1.5 mm, pitch 1.1, rotation time 0.5 s, transverse slice thickness 3 mm, matrix 512 × 512). Additional sagittal and coronal reformations in the lung window with a slice thickness of 3 mm were displayed for review.

### Image analysis

Two experienced radiologists in thoracic imaging (both 6 years) read all MDCT studies in consensus, as commonly done in this field [5, 6]. Image analyses were undertaken on a workstation with a picture archiving and communication system (Centricity™ Universal Viewer GE Healthcare, Chicago, Illinois). All CT studies were evaluated for the presence of distinctive lung pathologies including non-orthostatic reticulations, ground glass opacifications, consolidations, thickened interlobular septa, pleural and pericardial effusion, emphysema, honey combing, tree-in-bud sign, and pulmonary nodules and cysts, according to the glossary of the Fleischner Society [14]. In contrast to histologically proven pulmonary metastases, pulmonary nodules referred to the morphologic appearance of discrete rounded opacities up to 3 cm in diameter [14] without subsequent biopsy. Pulmonary nodules were followed-up according to current Fleischner guidelines [15]. Additionally, the maximum size of pulmonary nodules and cysts and the regional distribution were recorded for every patient.

### Genetic analysis

DNA was extracted from the blood of 57 patients (80%) using a QIAamp Blood Kit from Qiagen (Hilden, Germany). Mutation analysis was performed by direct sequencing of all 60 NF1 exons using a BigDye Sequencing kit as previously described [16, 17]. Pathogenicity of all mutations was defined according to the ACMG criteria for variant classification [18] and only pathologic mutations were evaluated statistically.

Genetically analyzed patients were categorized into the following mutation groups: (1) patients with large deletion covering the entire NF1 gene and several adjacent genes, (2) patients with intragenic NF1 mutations including nonsense, frameshift and canonical splice mutations, and (3) a further patient group without any specific gene mutations.

### Statistical analysis

Continuous variables are presented as mean ± SD. Categoric variables are presented with absolute and relative frequencies in percent. All variables were evaluated for normal distribution by the Shapiro–Wilk test. For pairwise comparisons of continuous normally distributed data, a t-test for independent samples was applied or otherwise a Mann–Whitney U rank sum tests. Categoric variables between different groups were compared by the χ^2^, Fisher exact test or Cochran-Armitage test for trend, when appropriate. Cramer’s V was calculated to measure the effect size for the χ^2^ tests. Values between 0.1 and 0.3 represented weak associations, whereas values 0.3–0.5 indicated medium associations between categorical parameters. Continuous variables between three groups were compared by one-way analysis of variance (ANOVA) for normal distributed data, or using Kruskal–Wallis test otherwise. Statistical significance was assumed for P values of less than 0.05 for simple inter-group comparisons or for P values of less than 0.017 for multiple comparisons (post-hoc analysis) after Bonferroni correction. Statistical analysis was performed using MedCalc 15.8 (MedCalc Software, Ostend, Belgium).

## Results

**Fig. 1:**
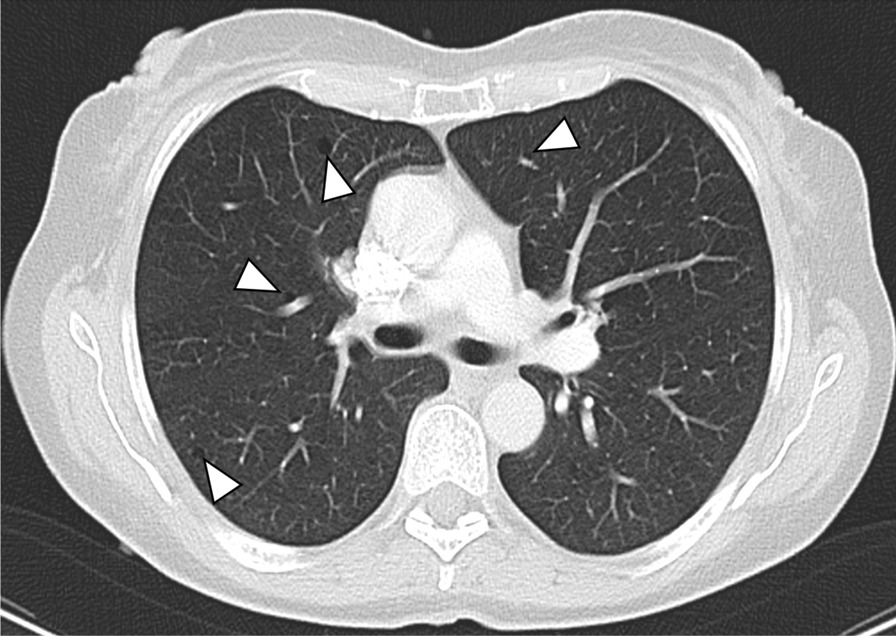
Transversal CT image of a 61-year old female non-smoker with several cysts in both upper lobes and the middle lobe (white arrowheads)

**Fig. 2:**
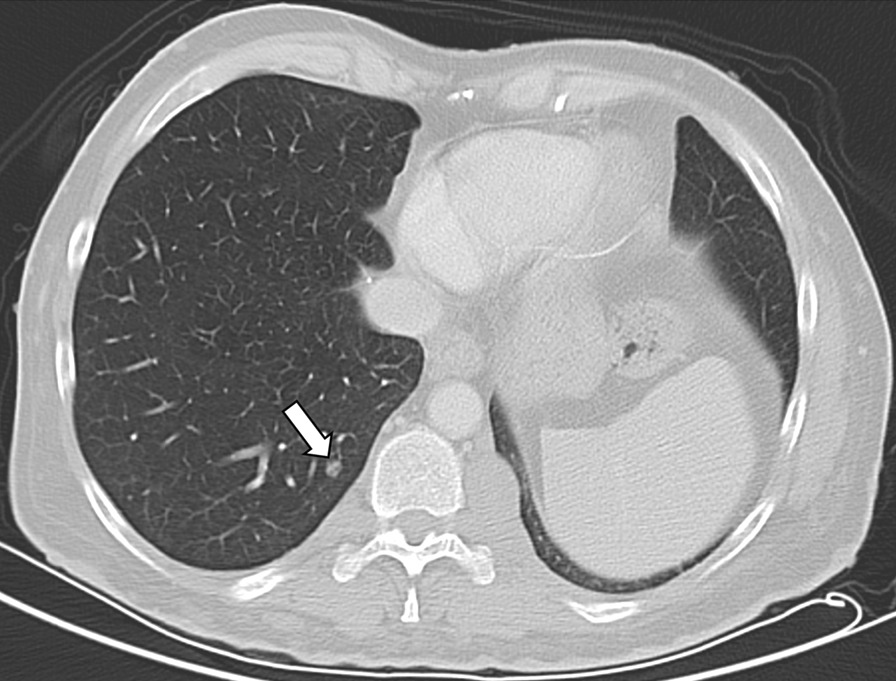
Transversal CT image of a 48-year old male non-smoker with a pulmonary nodule in the right lower lobe (white arrow)

**Fig. 3:**
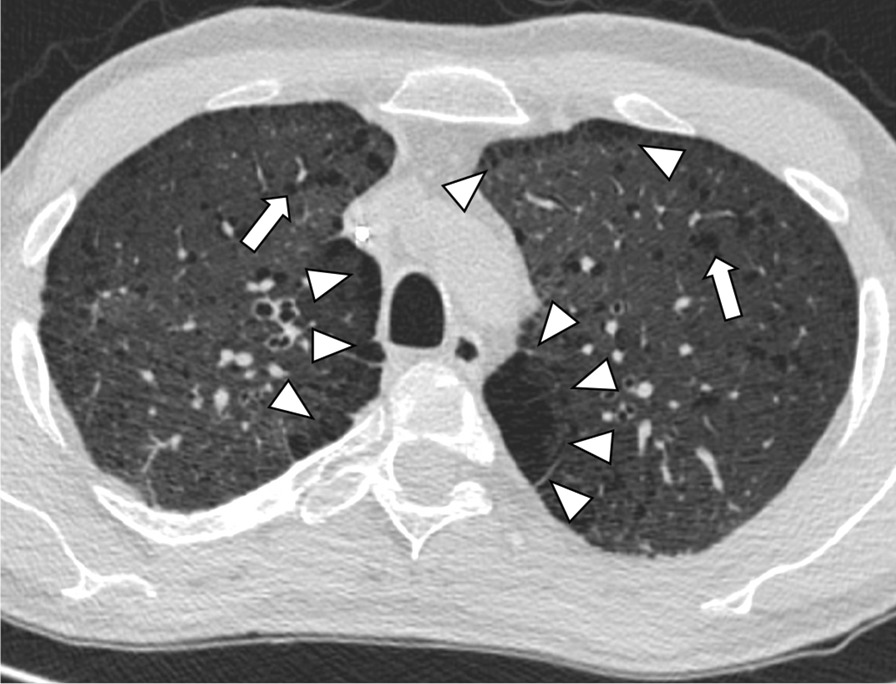
Transversal CT image of a 43-year old male smoker with extensive paraseptal emphysema in both apical upper lobes (white arrowheads) and multiple bilateral pulmonary cysts (white arrows)

Evaluation of the CT scans was feasible in all 71 patients (33 ± 14 years, range 2–68 years, 56% females). The characteristics of the study group are demonstrated in detail in Table 1. Seventeen patients (24%) were smokers and 36 patients (51%) were > 30 years old. MPNST was histologically proven in 30 patients (42%). In the total study group, pulmonary cysts (Fig. 1), intrapulmonary nodules (Fig. 2), and emphysema (Fig. 3) were the most common pulmonary findings with 35% (25/71), 32% (23/71), and 31% (22/71). The predominant localization of the cysts were the upper pulmonary lobes with 20%, followed by the lower lobes with 13% and the middle lobe with 2%. The mean diameter of the cysts was 8 ± 3 mm. Paraseptal emphysema was the predominant type of emphysema with 30% (21/71) followed by centrilobular emphysema with 7% (5/71). No panlobular emphysema, honey combing, tree-in-bud sign and pericardial effusion were present in the study population.

**Table 1:** Characteristics of the clinical and pulmonary findings in 71 NF1 patients

Characteristics	All NF1 patients n = 71
Female gender	40 (56)
Age (years ± SD)	33 ± 14
Current smokers	17 (24)
MPNST	30 (42)
Reticulations	18 (25)
GGO	7 (10)
Consolidation	6 (8)
Emphysema	22 (31)
Centrilobular	5 (7)
Paraseptal	21 (30)
Panlobular	0 (0)
Honey combing	0 (0)
Tree-in-bud sign	0 (0)
Nodules	23 (32)
≤ 10	18 (25)
> 10	5 (7)
Max. diameter (mm ± SD)	5 ± 2
Cysts	25 (35)
≤ 10	18 (25)
> 10	7 (10)
Max. diameter (mm ± SD)	8 ± 3
UL	14 (20)
ML	2 (3)
LL	9 (13)
Left	11 (15)
Right	14 (20)
Thickened interlobular septa	10 (14)
Pleural effusion	3 (4)
Pericardial effusion	0 (0)
Pulmonary metastasis	6 (8)

Data are presented as absolute numbers and frequencies (%) or as mean values ± standard deviation (SD)

*MPNST* malignant peripheral nerve sheath tumor, *GGO* ground glass opacity, *UL* upper lobe, *ML* middle lobe, *LL* lower lobe

**Fig. 4:**
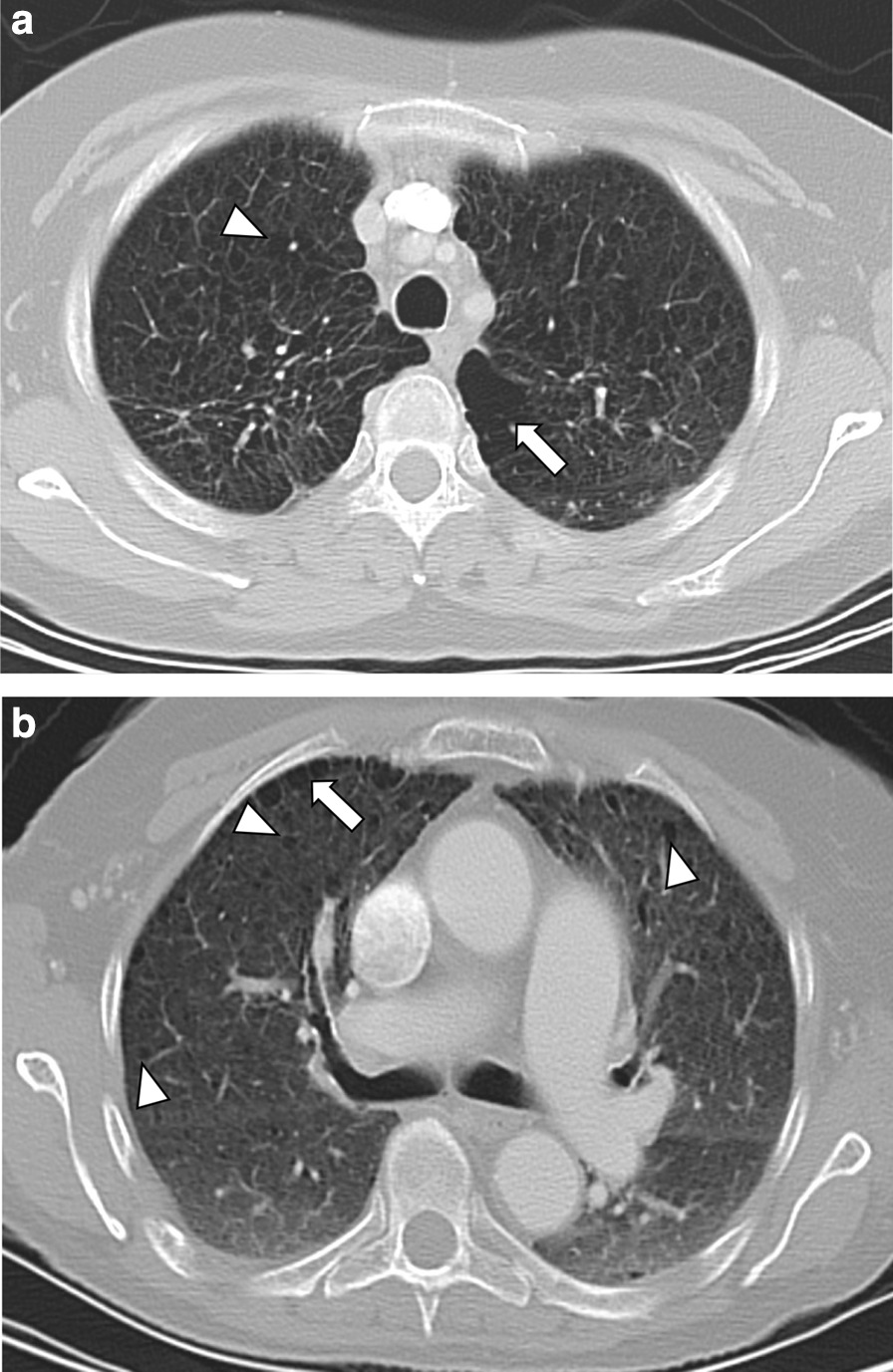
**a** Transversal CT image of a 52-year old female smoker with extensive centrilobular emphysema in both lung apices (white arrowheads) with bullae formation in the left apex (white arrow). **b** Transversal CT image of a 68-year old female non-smoker with a mild paraseptal emphysema (white arrow) and numerous pulmonary cysts in both upper lobes (white arrowheads)

**Fig. 5:**
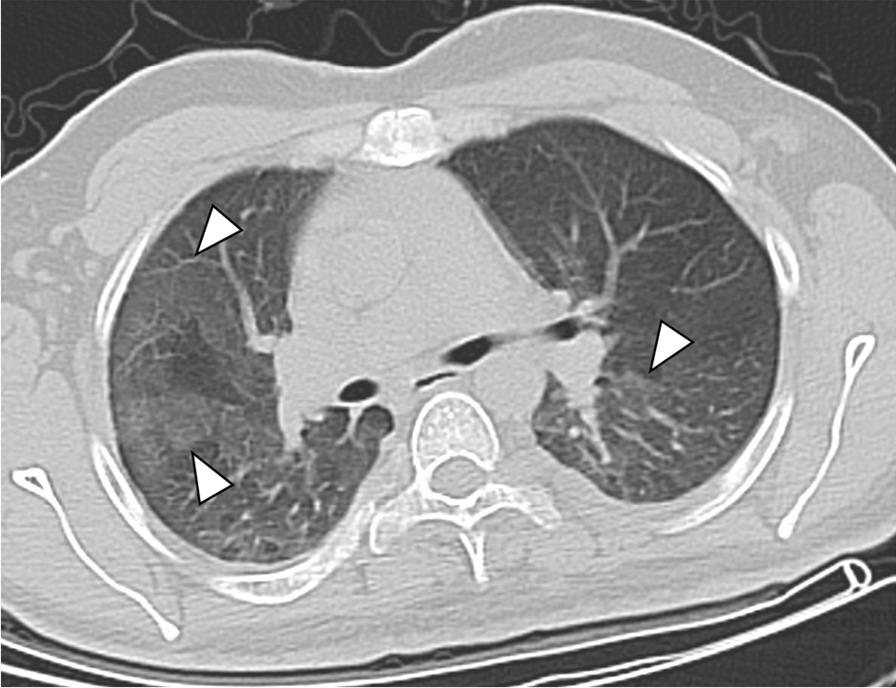
Transversal CT image of a 27-year old male non-smoker with predominantly right-sided ground glass opacifications (white arrowheads)

**Fig. 6:**
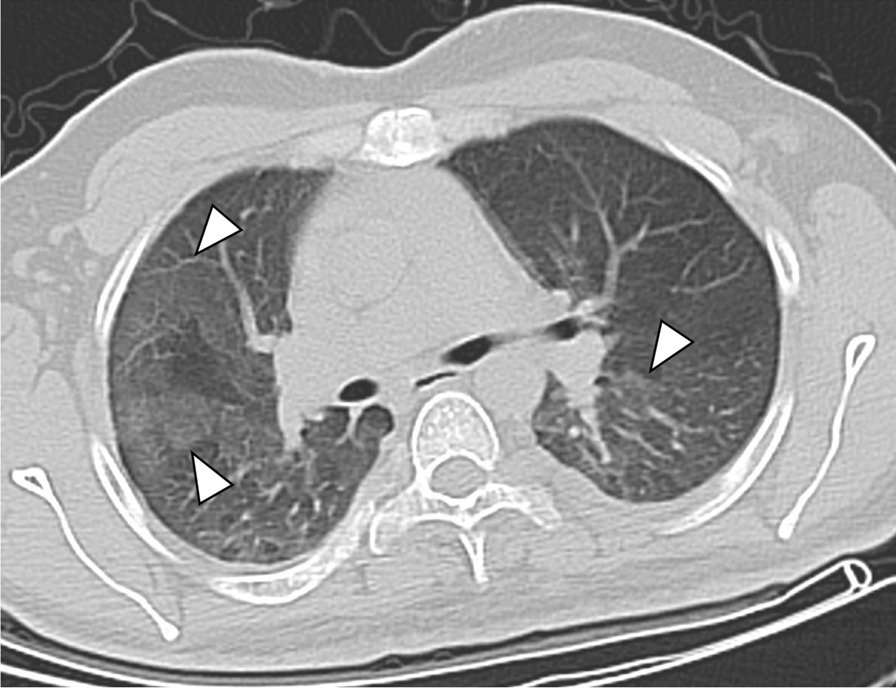
Transversal CT image of a 44-year old male smoker with interlobular septal thickening with reticular pattern in the left lower lobe (white arrowheads) as well as extensive paraseptal emphysema in the right lower lobe (white asterisk) and multiple bilateral cyst formation (white arrows)

Among never smokers, significantly more female patients were found compared to current smokers (67% vs. 29%, Table 2). With regard to intrapulmonary findings, a significantly higher number of histologically proven pulmonary metastases (24% vs. 2%) and MPNST (53% vs. 33%) was observed among current smokers compared to never smokers (Table 2). Moreover, centrilobular (Fig. 4a), but not paraseptal emphysema (Fig. 4b) was found significantly more often within the current smokers’ cohort compared to never smokers (24% vs. 2%, Table 2), indicating a medium association between smoking and the presence of centrilobular emphysema (Cramer’s V = 0.363). A trend towards multiple pulmonary cysts (24% vs. 7%, *p* = 0.08), predominantly located in the upper lobes was seen within the current smokers’ group (Table 2). Other pulmonary findings like ground glass opacifications (Fig. 5), reticulations (Fig. 6) or consolidations did not differ significantly between patients with or without a history of smoking.

**Table 2:** Comparison of the clinical and pulmonary findings between current smokers, previous smokers and never smokers

	Current smokers n = 17	Previous smokers^a^ n = 8	Never smokers n = 46	*p* value	Association by Cramer’s V
Female gender	5 (29)	4 (50)	31 (67)	**< 0.05**	0.323
Age	31 ± 11	42 ± 7	35 ± 16	0.08	–
MPNST	9 (53)	6 (75)	15 (33)	**< 0.05**	0.292
Reticulations	4 (24)	2 (25)	12 (26)	0.98	–
Nodules	8 (47)	2 (25)	13 (28)	0.33	–
≤ 10	6 (35)	1 (12)	11 (24)	0.44	–
> 10	2 (12)	1 (12)	2 (4)	0.48	–
Max. diameter (mm)	6 ± 6	6 ± 2	5 ± 2	0.69	–
GGO	3 (18)	0 (0)	4 (9)	0.35	–
Consolidation	1 (6)	0 (0)	5 (11)	0.54	–
Emphysema	8 (47)	2 (25)	12 (26)	0.26	–
Centrilobular	4 (24)	0 (0)	1 (2)	**< 0.05**	0.363
Paraseptal	8 (47)	2 (25)	11 (24)	0.19	–
Cysts	7 (41)	2 (25)	16 (35)	0.73	–
≤ 10	3 (18)	2 (25)	13 (28)	0.69	–
> 10	4 (24)	0 (0)	3 (7)	0.08	–
Max. diameter (mm)	9 ± 4	8 ± 2	6 ± 2	0.29	–
UL	7 (41)	0 (0)	7 (15)	**< 0.05**	0.325
ML	0 (0)	0 (0)	2 (4)	0.57	–
LL	0 (0)	2 (25)	7 (15)	**< 0.05**	0.310
Thickened interlobular septa	2 (12)	2 (25)	6 (13)	0.64	–
Pleural effusion	0 (0)	1 (12)	2 (4)	0.35	–
Pulmonary metastasis	4 (24)	1 (12)	1 (2)	**< 0.05**	0.325

Data are presented as absolute numbers and frequencies (%) or as mean values ± standard deviation (SD)

^a^Smoking cessation > 5 years ago

Cochran-Armitage test for trend *p* values < 0.05 (**bold**) were considered to indicate statistical significance

*MPNST* malignant peripheral nerve sheath tumor, *GGO* ground glass opacity, *UL* upper lobe, *ML* middle lobe, *LL* Lower lobe

Only in adult patients over 18 years pulmonary cysts were observed, whereas all other pulmonary findings did not differ significantly between the different age groups (Table 3).

**Table 3:** Comparison of the clinical and pulmonary findings between children (< 12 years), adolescents (12–18 years) and adults (> 18 years)

	Children (< 12 years) n = 4	Adolescents (12–18 years) n = 5	Adults (> 18 years) n = 62	*p* value^a^	Association by Cramer’s V
Female gender	2 (50)	3 (60)	35 (56)	0.88	–
Age	7 ± 4*	16 ± 1*	36 ± 12	**< 0.001**	–
Current smokers	0 (0)	1 (20)	16 (26)	0.25	–
MPNST	0 (0)	2 (40)	28 (45)	0.10	–
Reticulations	1 (25)	0 (0)	17 (27)	0.49	–
Nodules	1 (25)	1 (20)	21 (34)	0.74	–
≤ 10	1 (25)	1 (20)	16 (26)	0.87	–
> 10	0 (0)	0 (0)	5 (8)	0.41	–
Max. Diameter (mm)	2	3	11	0.37	
GGO	0 (0)	0 (0)	7 (11)	0.32	–
Consolidation	1 (25)	0 (0)	5 (8)	0.45	–
Emphysema	0 (0)	1 (20)	21 (34)	0.13	–
Centrilobular	0 (0)	0 (0)	5 (8)	0.41	–
Paraseptal	0 (0)	1 (20)	20 (34)	0.15	–
Cysts	0 (0)	0 (0)	25 (40)	**< 0.05**	0.281
≤ 10	0 (0)	0 (0)	18 (29)	0.08	–
> 10	0 (0)	0 (0)	7 (11)	0.32	–
Max. Diameter (mm)	0	0	6 ± 3	–	–
UL	0 (0)	0 (0)	14 (23)	0.13	–
ML	0 (0)	0 (0)	2 (3)	0.61	–
LL	0 (0)	0 (0)	9 (15)	0.25	–
Thickened interlobular septa	0 (0)	0 (0)	10 (16)	0.22	–
Pleural effusion	0 (0)	0 (0)	3 (5)	0.53	–
Pulmonary metastasis	0 (0)	1 (20)	5 (8)	0.93	–

Data are presented as absolute numbers and frequencies (%) or as mean values ± standard deviation (SD)

^a^Fisher exact test; Cochran-Armitage test for trend: *p* values < 0.05 (**bold**) were considered to indicate statistical significance. *p values < 0.017 versus Adults, applying Bonferroni correction for multiple comparisons across the three groups

*MPNST* malignant peripheral nerve sheath tumor, *GGO* ground glass opacity, *UL* upper lobe, *ML* middle lobe, *LL* lower lobe

As expected, pulmonary metastasis were only found in patients with histologically proven MPNST (21% vs. 0%, *p* < 0.05, Table 4, Fig. 5). The presence (57% vs. 15%, *p* < 0.001) and the number of pulmonary nodules up to 3 cm maximum diameter were significantly higher in the MPNST group (for both ≤ 10 nodules and > 10 nodules, *p* < 0.05, Table 4). All other pulmonary findings were comparable between both investigated subgroups (*p* > 0.05 for all variables).

**Table 4:** Comparison of the clinical and pulmonary findings between patients with and without MPNST

	MPNST n = 30	No MPNST n = 41	*p* value^a^
Female gender	12 (40)	28 (68)	**< 0.05**
Age	36 ± 13	32 ± 15	0.26
Current Smokers	9 (30)	8 (20)	0.46
Reticulations	10 (33)	8 (20)	0.30
Nodules	17 (57)	6 (15)	**< 0.001**
≤ 10	12 (40)	6 (15)	**< 0.05**
> 10	5 (17)	0 (0)	**< 0.05**
Ø Diameter (mm)	5 ± 2	4 ± 2	0.23
GGO	4 (14)	3 (7)	0.42
Consolidation	3 (11)	3 (7)	0.67
Emphysema	11 (37)	11 (27)	0.53
Centrilobular	2 (7)	3 (7)	1.0
Paraseptal	11 (37)	10 (24)	0.39
Cysts	10 (33)	15 (37)	0.97
≤ 10	8 (27)	10 (24)	0.95
> 10	2 (7)	5 (12)	0.69
Ø Diameter (mm)	8 ± 2	7 ± 4	0.59
UL	6 (20)	8 (20)	0.80
ML	1 (3)	1 (2)	1.0
LL	3 (10)	6 (15)	0.72
Thickened interlobular septa	5 (17)	5 (12)	0.73
Pleural effusion	2 (7)	1 (2)	0.57
Pulmonary metastasis	6 (21)	0 (0)	**< 0.05**

Data are presented as absolute numbers and frequencies (%) or as mean values ± standard deviation (SD)

^a^Fisher exact test or χ^2^ test; *p* values < 0.05 (**bold**) were considered to indicate statistical significance

*MPNST* malignant peripheral nerve sheath tumor, *GGO* ground glass opacity, *UL* upper lobe, *ML* middle lobe, *LL* lower lobe

From 57 patients (80%) with available genetic analyses, no specific genetic mutations were observed in 13 patients (23%). Large deletion of the entire NF1 gene occurred in 8 patients (14%), while in 31 patients (54%) intragenic NF1 mutations were observable, including nonsense (n = 13), frameshift (n = 12) and canonical splice mutations (n = 6). In the remaining 5 patients silent mutations of uncertain significance did occur (intronic mutation (n = 2), intronic deletion (n = 2), constrained canonical splicing (n = 1)), which were excluded from further statistical analysis as is presented in the Additional Table 1 in more detail (see Additional file 1). However, no significant difference between the abovementioned mutation subgroups could be observed for any pulmonary finding (*p* > 0.05 for all comparisons). Moreover, significantly more current smokers were found among those patients, who didn’t have any specific genetic mutation compared to patients with large deletions and intragenic mutations (38% vs. 0% vs. 3%, *p* < 0.05). Ten patients did not have any pulmonary abnormalities, of whom 4 patients did not show any specific genetic mutations, 2 patients had a frameshift mutation, while the remaining 4 patients had a point mutation. None of the patients without pulmonary abnormalities revealed chromosomal deletions.

## Discussion

In the present study we evaluated several pulmonary findings in a single-center cohort of patients with NF1 with regard to their smoking history, age, specific genetic mutations, and the presence of MPNST. The most commonly found pulmonary findings among NF1 patients were pulmonary cysts (35%), intrapulmonary nodules (32%), and paraseptal emphysema (30%), which all occurred independent from the investigated genetic aberrations. Our results demonstrated an association of presence of pulmonary metastasis, MPNST and centrilobular emphysema with smoking. Furthermore, we found a linkage between adulthood and the presence of pulmonary cysts, which were not found among adolescents or children. The presence of MPNST did not influence any pulmonary findings besides the presence and number of pulmonary nodules.

Our most commonly found abovementioned pulmonary findings as well as the predominance of pulmonary cysts in the upper lung lobes and the absence of pulmonary fibrosis in terms of honey combing are consistent with previous results [3, 7–9]. Alike the results of Ueda et al. [9], we could not find a significant difference between the presence of cysts and their maximum size between current smokers, previous smokers and never smokers, thereby supporting the thesis of its independent occurrence from smoking [7–9]. However, our results demonstrated significantly higher number of cysts in upper lobe location in current smokers compared with previous and never smokers, which may be caused by the relatively overventilation of the lung apices compared to the lung bases, which are prone to smoke-related inhalation injury [19]. Furthermore, both current as well as previous smokers revealed significantly higher numbers of pulmonary metastases compared with non-smokers and MPNST, which might be explained by the proto-oncogenic stimulation caused by smoking [20] and by the fact, that smoking is regarded as risk factor for pulmonary metastasis in gastrointestinal cancers [21, 22] and breast cancer [23]. Moreover, it has been suggested, that NF1 may enhance the pulmonary sensitivity to cigarette smoke [7].

However, none of the investigated pulmonary pathologies were associated with specific genetic mutations, indicating that many types of NF1 mutations can lead to a variety of different pulmonary manifestations of NF1. These results are in line with previous findings in NF1 patients with plexiform neurofibromas (PNF), where no correlation could be found between the type of the specific NF1 mutation and size, location or feature of the PNFs [16].

Our data additionally suggest the occurence of pulmonary cysts only in adult patients, which were mostly located in the upper lobes. These results are supported by previous findings in elderly asymptomatic individuals, according to which pulmonary cysts were found significantly more often in the elderly individuals over 75 years compared with individuals under 55 years (25% vs. 0%, *p* = 0.02) independent of smoking history [24]. It may be hypothesized that the same factors like the lymphoplasmocytic inflammation of the alveolar septa that have been well documented in amyloidosis from surgical lung biopsy specimens [25] leading to bronchial obstruction and consequent cyst formation are age-dependent and may also influence cyst development in the healthy elderly individuals and in NF1 patients, however, at an earlier stage.

Interestingly, only the centrilobular, but not the paraseptal emphysema was increasingly found in smokers. This partly opposes the results of Ueda et al. [9], who found a strong dependence of emphysema and smoking. However, the colleges described the emphysema in an upper and peripheral dominant distribution without mentioning its morphologic subtype on CT [24–26]. Centrilobular emphysema usually occurs in the upper or apical lung lobes, might be located centrally and peripherally in the lungs with increasing severity [26, 27], and is strongly associated with smoking [25–29] in contrast to paraseptal emphysema [30]. Therefore, Ueda et al. might have observed predominantly advanced emphysema of the centrilobular type within their subgroup of smokers. This hypothesis is also supported by a case report of apically located, combined paraseptal and panlobular emphysema in a non-smoking NF1 patient [31]. This finding is of great importance due to the fact, that although paraseptal emphysema increases the risk of spontaneous pneumothorax formation [32, 33], this phenomenon was almost always seen in smokers. Smoking was shown to increase the risk of a first spontaneous pneumothorax by ninefold in women and 22-fold in men and there was a strong relationship between the level of smoking and the occurrence of a spontaneous pneumothorax [34]. Moreover, in contrast to centrilobular emphysema, paraseptal emphysema was not associated with increased dyspnea or an impairment of the pulmonary function in a large multi-center study [30]. Hence, a smoking cessation might be the best preventive strategy for those NF1 patients with paraseptal emphysema.

The presence of MPNST did not affect any pulmonary findings except for the presence and number of pulmonary nodules and pulmonary metastasis. As the mean maximum diameter of the nodules of patients with MPNST was only 5 ± 2 mm, a short-term follow-up was recommended in the majority of cases, according to the Fleischner Guidelines 2017 [15]. Therefore, a malignant etiology of several pulmonary nodules could not been excluded and may represent the main reason for overrepresentation in patients with MPNST.

A comparison of our findings with prior cohorts is warranted, for example, the systematically studied NF1 population accrued in the Baylor College of Medicine Neurofibromatosis Program (BNFP) from 1978 to 1986 [35, 36]. In terms of their lungs, those subjects, ranging in age from infancy to elderly, had their histories taken and physical examinations done by one person and subjected to a routine chest X-ray. In short, a thorough, reliable medical history and routine physical examination, supplemented by a routine chest X-ray, was inadequate to identify the lung pathology findings identified here. In order to identify a satisfactory estimate of the range of the lung’s NF1 features, consequences and complications, a far more intense and focused pulmonary evaluation using an MDCT is necessary [8, 9]. Potential elements of that focus are established herein.

Our study had several limitations. First, we didn’t perform high-resolution CT (HRCT) in our study, which might have affected our results. However, due to the long period of our data collection between 2006 and 2018 and due to the retrospective study design, no HRCT was available for the complete study cohort. Nevertheless, all reconstructions were performed with a slice thickness of 3 mm and were additionally regarded in every patient to increase the yield of manifest pulmonary findings by proving the appearance of them in different orientations. Furthermore, all studies were performed on the same PET/CT scanner, thereby increasing the homogeneity of the interpretation of the results. A second limitation refers to the specific mutation analysis, which was available in a subgroup of 57/71 NF1 patients (80%). However, the analysis included large deletions covering the entire NF1 gene and several adjacent genes and various intragenic NF1 mutations including nonsense, frameshift and canonical splice mutations.

A third limitation is the missing information on the package years to further quantify the impact of smoking on pulmonary pathologies due to the retrospective study design. Therefore, the present results should be interpreted with caution and should be verified by larger prospective trials using uniform HRCT protocols together with pulmonary function tests as well as follow-up data to prove disease progression and further evaluate the clinical relevance of different pulmonary findings in NF1 patients.

## Conclusion

Independent of the specific subtype of genetic mutation of the NF1 gene, a variety of different pulmonary pathologies may occur in NF1 patients, of which the major three findings were pulmonary cysts, intrapulmonary nodules, and paraseptal emphysema. The presence of pulmonary metastases and MPNST was significantly higher not only in current, but also in previous smokers compared to never smokers. Moreover, centrilobular emphysema was associated with persistent smoking, indicating the value of smoking secession in smoking NF1 patients and the strong advice not to start smoking in never smoking NF1 patients as possible preventive strategy for clinicians. In clinical routine MDCT instead of a chest X-ray combined with a comprehensive medical history of pulmonary symptoms and physical examination is mandatory for reliable detection of various pulmonary manifestations in NF1 patients.

## Supplementary Information

**Additional file 1**. Additional Table 1.

## Abbreviations

BNFP: Baylor College of Medicine Neurofibromatosis Program
CT: Computed tomography
FOV: Field of view
GGO: Ground glass opacity
HRCT: High-resolution CT
ILD: Interstitial lung disease
LL: Lower lobe
MDCT: Multidetector computed tomography
ML: Middle lobe
MPNST: Malignant peripheral nerve sheath tumor
MPR: Multiplanar reconstruction
NIH: National Institutes of Health
NF1: Neurofibromatosis type 1
PNF: Plexiform neurofibroma
SD: Standard deviation
UL: Upper lobe

## Available data and materials

The datasets used and/or analyzed during the current study are available from the corresponding author (MA) on reasonable request.
