# Supplementary material for: Structural alteration of lung parenchyma in patients with NF1: a phenotyping study using multidetector computed tomography (MDCT)
Source: Orphanet J Rare Dis. 2021 Jan 14;16:29. doi: 10.1186/s13023-021-01672-0 (PMC7809820; doi:10.1186/s13023-021-01672-0)
Supplement: Supplementary file 1 — Additional file 1. Additional Table 1. [file 13023_2021_1672_MOESM1_ESM.docx]

# Additional Material

**Additional Table 1. Influence of pathologic genetic mutations on the occurrence of pulmonary findings**

|  | **Large deletion of entire NF1 gene**  **n=8** | **Intragenic NF1 mutations ***  **n=31** | **No Mutation**  **n=13** | **P-value**^a^ |
| --- | --- | --- | --- | --- |
| Female gender | 4 (50) | 17 (55) | 6 (46) | 0.86 |
| Age | 31±12 | 34±14 | 26±10 | 0.20 |
| Current Smokers | 0 (0) | 1 (3) | 5 (38) | **<0.05**^#^ |
| MPNST | 4 (50) | 6 (19) | 4 (31) | 0.21 |
| Reticulations | 3 (38) | 9 (29) | 2 (15) | 0.50 |
| Nodules  ≤10  >10  Max. diameter (mm) | 2 (25)  1 (13)  1 (13)  7 | 11 (35)  10 (32)  1 (3)  10 | 3 (23)  1 (8)  2 (15)  8 | 0.67  0.16  0.33  - |
| GGO | 1 (13) | 2 (6) | 1 (8) | 0.85 |
| Consolidation | 0 (0) | 3 (10) | 0 (0) | 0.34 |
| Emphysema  -centrilobular  -paraseptal | 4 (50)  0 (0)  4 (50) | 9 (29)  1 (3)  9 (29) | 2 (15)  1 (8)  2 (15) | 0.24  0.65  0.24 |
| Cysts  ≤10  >10  Max. Diameter (mm)  UL  ML  LL | 1 (13)  1 (13)  0 (0)  4  1 (13)  0 (0)  0 (0) | 13 (42)  11 (35)  2 (6)  12  6 (19)  1 (3)  6 (19) | 3 (23)  2 (15)  1 (8)  6  2 (15)  0 (0)  1 (8) | 0.20  0.24  0.74  -  0.88  0.71  0.28 |
| Thickened interlobular septa | 1 (13) | 5 (16) | 0 (0) | 0.31 |
| Pleural effusion | 0 (0) | 2 (6) | 0 (0) | 0.49 |

*5 Patients with mutations of uncertain significance were excluded. MPNST: Malignant peripheral nerve sheath tumor; GGO: Ground glass opacity; UL: Upper lobe; ML: Middle lobe; LL: Lower lobe. ^a^Fisher exact test or ꭓ² test; ^#^p values <0.05 (**bold**) were considered to indicate statistical significance
